# Malaria drives T cells to exhaustion

**DOI:** 10.3389/fmicb.2014.00249

**Published:** 2014-05-27

**Authors:** Michelle N. Wykes, Joshua M. Horne-Debets, Chiuan-Yee Leow, Deshapriya S. Karunarathne

**Affiliations:** ^1^Molecular Immunology Laboratory, QIMR Berghofer Medical Research InstituteBrisbane, QLD, Australia; ^2^The School of Medicine, University of QueenslandBrisbane, QLD, Australia

**Keywords:** CD8^+^ T cell, malaria, exhaustion, PD-1, chronic disease, CD4^+^ T cells, B cells, PD-L1

## Abstract

Malaria is a significant global burden but after >30 years of effort there is no vaccine on the market. While the complex life cycle of the parasite presents several challenges, many years of research have also identified several mechanisms of immune evasion by *Plasmodium* spp. Recent research on malaria, has investigated the programmed cell death-1 (PD-1) pathway which mediates exhaustion of T cells, characterized by poor effector functions and recall responses and in some cases loss of the cells by apoptosis. Such studies have shown exhaustion of CD4^+^ T cells and an unappreciated role for CD8^+^ T cells in promoting sterile immunity against blood stage malaria. This is because PD-1 mediates up to a 95% reduction in numbers and functional capacity of parasite-specific CD8^+^ T cells, thus masking their role in protection. The role of T cell exhaustion during malaria provides an explanation for the absence of sterile immunity following the clearance of acute disease which will be relevant to future malaria-vaccine design and suggests the need for novel therapeutic solutions. This review will thus examine the role of PD-1-mediated T cell exhaustion in preventing lasting immunity against malaria.

## INTRODUCTION

Malaria is a mosquito-borne infectious disease of humans caused by parasitic protozoans of the genus *Plasmodium.* There are five species that infect humans and annual deaths from the malaria parasite *Plasmodium falciparum* (*Pf*) alone are estimated at 780,000, with approximately 225 million clinical infections worldwide (Schwartz et al., 2012). The *Anopheles* mosquito, serves as a transmission vector for the parasite. An infected mosquito introduces sporozoites into the blood or lymph of bitten individuals when taking a blood meal. These sporozoites then migrate to the host’s liver, where they begin to invade and multiply. During the first week of infection, sporozoites develop into merozoites and then exit the hepatocytes to invade red blood cells progressively, resulting in massive destruction of red blood cells. This eventually leads to anemia in infected subjects due to the loss of erythrocyte and severe symptoms associated with malaria.

Over the last 10 years, more than 40 vaccines were developed to control the morbidity of malaria which then reached the clinical trial stage (Schwartz et al., 2012). Most of these vaccines were specifically designed to target liver or blood stage parasites, the majority through protection by antibodies and/or CD4^+^ T cells. Although some of them showed potent protection at pre-clinical trials (Schwartz et al., 2012), none to date conferred complete protection against both clinical and severe malaria. The only vaccine to reach stage 3b, RTS,S/AS01E, showed approximately 50% efficacy for 18 months (Alonso et al., 2005) but immunity was significantly diminished within 4 years (Olotu et al., 2013). Notably, protection declined over time and with increasing malaria exposure.

To explain why these vaccines have been unsuccessful, it is important to understand natural immunity against malaria. Perhaps most informative has been an intensive longitudinal study of immunity in Malian children and adults (Tran et al., 2013). In this study, 251 healthy children and adults who were free of blood-stage *Plasmodium* infections, by polymerase chain reaction (PCR), were enrolled just prior to an intense 6-month malaria season. Subsequent clinical malaria episodes were detected by weekly active surveillance or self-referral. Asymptomatic infections were detected by blood-smear microscopy and PCR analysis of blood collected every 2 weeks for 7 months. These studies found that while the risk of symptomatic malaria decreased with increasing age, the risk *P. falciparum* infections did not change. This means that despite years of exposure to intense *Pf* transmission, there was no evidence of acquired, sterile immunity to the parasite in this population, even as clinical immunity to blood-stage malaria was clearly acquired (Tran et al., 2013). Significantly, this indicates that while anti-malarial immune responses to protect against symptoms were effectively induced, sterile immunity against the parasite was not acquired in the same time frame.

## NATURALLY ACQUIRED IMMUNITY

The factors that regulate anti-parasite immunity (i.e., those processes that kill parasites and reduce parasite biomass in the body) are not completely understood. For decades it has been known that antibodies play an important role in protection against malaria, and it has been assumed that antibody responses alone can provide sufficient protection to control this disease. This was predominantly based on studies where the transfer of IgG from malaria-immune individuals to non-immune individuals markedly reduced parasite burden (Cohen et al., 1961). Studies indicating that antibodies protect against malaria have led to a large-scale effort to develop a B cell targeted vaccine against malaria, with limited success to date. In endemic settings, memory antibody responses against *Plasmodium* parasites are short-lived and require constant parasite exposure (Crompton et al., 2010). Furthermore, a year-long study in malaria-endemic Mali, found that both *Pf*-specific memory B cells (MBC) and antibody titers increased after acute malaria and then, after 6 months of decreased *Pf* exposure, contracted to a point slightly higher than pre-infection levels (Weiss et al., 2010). Further, the 19-kDa carboxyl-terminal fragment of merozoite surface protein 1 (MSP1_19_) was a leading malaria vaccine candidate, tested in Kenya which has holoendemic transmission of *Pf.* In these trials, vaccination with MSP1_19_ generated very high titers of antibodies, but did not protect against infection (Ogutu et al., 2009). An evaluation of this vaccine using an experimental mouse model found that vaccination with MSP1_19_ generated MSP1_19_-specific MBC capable of secreting antibodies in response to the vaccine (MSP1_19_), but not infection (Wykes et al., 2005). In fact, infection caused apoptosis of MBC. Subsequent studies showed malarial infections result in a decrease in the proportion of dendritic cells (DCs) that expressed the B-cell survival factor, BAFF, resulting in a decreased ability of these DCs to support MBC survival (Liu et al., 2012). A review proposes a model of how the parasite may mediate these effects by direct interaction with B cells and modulation of the host’s BAFF-immune pathway (Scholzen and Sauerwein, 2013).

CD4^+^ T cells consist of several helper-subtypes which shape immune responses against particular pathogens. During malaria, CD4^+^ T cell subsets have multiple roles in protection, pathogenesis and also escape from immune responses. CD4^+^ T cells have been demonstrated to be the major source of both interferon-γ (IFN-γ) and tumor necrosis factor alpha (TNF-α) during experimental malaria in mice (Muxel et al., 2011) which are implicated in both protection and pathology of this disease. Studies in mice infected with *Plasmodium chabaudi* malaria have shown that IFN-γ and TNF-α cooperatively induce nitric oxide synthase expression in the spleen to control peak parasite burden (Jacobs et al., 1996). Similarly, in humans, early IFN-γ responses to *Pf* correlate with better anti-parasite immunity (McCall et al., 2010). IFN-γ contributes to a vast network of protective responses against malaria, summarized in McCall and Sauerwein (2010). Of particular note is a study which investigated the effects of chronic malaria on MSP1-specific transgenic CD4^+^ T cells (Stephens and Langhorne, 2010). These parasite-specific T cells were seeded into Thy1.1 congenic mice which were then infected with 10^5^ *P. chabaudi* infected red cells. One half of the mice were treated with Chloroquine on days 30–34 to clear chronic malaria. After 60 days, flow cytometric analysis of transgenic T cells found that approximately 25% of memory CD44^+^IL-7R^+^ CD4^+^ T cells were lost in untreated mice compared to drug-treated mice which had cleared the infection (Stephens and Langhorne, 2010). This study highlights that ongoing infections cause a loss of some parasite-specific memory T cells capable of protection from re-infection.

The loss of CD4^+^ T cells during malaria was perhaps first suggested when up to 99% of parasite-specific T cells labeled with the fluorescent dye 5-(and -6)-carboxy-fluorescein succinimidyl ester (CSFE), were found to be deleted only following infection of mice (Hirunpetcharat and Good, 1998). Subsequent studies also found deletion of T cells specific for the malaria vaccine, MSP1_19_ during malaria infections (Wipasa et al., 2001). Further studies excluded a role for TNF or Fas pathways but implicated IFN-γ in loss of these cells (Xu et al., 2002). However, FOXP3-expressing CD4^+^CD25^+^ regulatory T cells were also shown to correlate with more rapid parasite growth in human malaria infections (Walther et al., 2005) which may explain why protective cells subside. Mouse models also showed IL-10 produced by these cells was responsible for poor immunity (Couper et al., 2008). Finally, other studies identified elevated numbers of a highly suppressive subset of regulatory T cells in patients with severe malaria (Minigo et al., 2009).

The role of CD8^+^ T cell-mediated immunity against blood-stage malaria has been largely overlooked, with research being limited to protection against liver-stage infection, the pathogenesis of cerebral malaria (Hafalla et al., 2006) and damage to splenic architecture (Beattie et al., 2006). This view was adopted because parasites and infected erythrocytes have no known antigen presentation machinery and therefore T cells supposedly cannot interact directly with these potential targets (Langhorne et al., 2008). However, early studies in experimental animal models found that depletion of CD8^+^ T lymphocytes during blood stage *P. chabaudi* infections, significantly delayed clearance of the infection (Podoba and Stevenson, 1991). Other groups showed that naive mice, transfused with CD8^+^ T cells derived from mice that survived two successive bouts of lethal *P. yoelii* infections with drug cure, subsequently survived lethal blood stage malaria challenge (Imai et al., 2010). More recent studies in experimental mouse models have shown that malaria parasites can parasitize erythroblasts, which have the capacity to activate CD8^+^ T cells (Imai et al., 2013). What has remained unclear is that while these studies showed CD8^+^ T cells had potential to protect against blood stage malaria, direct studies did not find a clear role for these cells in protection against blood stage disease.

## PROGRAMMED CELL DEATH 1

Programmed cell death-1 (PD-1) is a member of the extended family of molecules that are known to down-regulate T cell function. PD-1 has two known ligands, PD-L1 (B7-H1; Dong et al., 1999; Freeman et al., 2000) and PD-L2 (B7-DC; Latchman et al., 2001; Tseng et al., 2001), which both belong to the B7 co-signaling molecule family. Expression of PD-1 can be observed on T cells, B cells, natural killer T cells, DCs, and activated monocytes (Keir et al., 2008). PD-1 is not expressed on resting T cells but is inducible upon activation (Agata et al., 1996). Functional effects of PD-1 ligation can be observed within a few hours after T cell activation but PD-1 cell surface protein up-regulation requires 24 h (Chemnitz et al., 2004). When PD-1 is engaged simultaneously with T cell receptor signals, it can trigger an inhibitory signal, although no signal transduction occurs when PD-1 is cross-linked alone (Sharpe et al., 2007). In general, interactions between PD-1 on T cells and its ligand, PD-L1, control the induction and maintenance of peripheral T-cell tolerance and negatively regulate proliferation and cytokine production by T cells during immune responses to pathogens or cancer (Sharpe et al., 2007).

A main feature of potent immunity against intracellular pathogens is the development of an optimal T cell response which shows rapid proliferative potential, low apoptosis and polyfunctionality (Lukens et al., 2008). During acute infections, optimal functioning T cells clear the pathogen, eventually leading to development of robust memory T cells. Those T cells have the ability to mount rapid recall response and re-establish polyfunctional effector mechanisms upon antigen re-exposure (Wherry, 2011). T cell exhaustion is defined by inferior effector function, sustained expression of inhibitory receptors (such as PD-1), poor recall responses and a transcriptional state distinct from that of functional effector or memory T cells (Wherry, 2011). In many chronic infectious diseases, antigen specific T cells become functionally impaired or exhausted. For example, viruses, such as human immunodeficiency virus (HIV) and hepatitis C virus (HCV) have been shown to induce T cell exhaustion mediated by PD-1 (Day et al., 2006; Urbani et al., 2006). Significantly, blockade of the PD-1 pathway improved immunity (Freeman et al., 2006; Urbani et al., 2008). PD-1-related exhaustion has also been implicated in other chronic protozoan infections, such as Leishmaniasis (Liang et al., 2006; Joshi et al., 2009). Further, the apicomplexan parasite, *Toxoplasma gondii* has been shown to induce PD-1 expression on CD8^+^ T cells, but function was restored by experimental blockade of the PD-1 pathway during chronic murine infections (Bhadra et al., 2011). Together these data have established T cell exhaustion by PD-1 as a key mechanism in chronicity of infectious diseases.

## EXHAUSTION OF CD4^+^ T CELLS DURING MALARIA

PD-1 has been implicated in the pathogenesis of malaria. One of the first studies to examine PD-1 expression during malaria used a mouse model to show PD-1 expression on IL-7R^lo^-expressing CD4^+^ and CD8^+^ T cells (Chandele et al., 2011). These PD-1-expressing cells (especially CD8^+^ T cells) were almost completely lost within 30 days of infection (Chandele et al., 2011). The study did not however measure functional responses to identify T cell exhaustion. Similarly, subsequent studies showed that PD-1 was also expressed on CD4^+^ (Butler et al., 2012; Illingworth et al., 2013) and CD8^+^ T cells (Illingworth et al., 2013) in blood of *Pf*-infected individuals in Mali and Kenya, but no functional evidence of exhaustion was provided.

To validate these observations, a murine model of blood stage malaria was adopted to explore the effects of increased expression of PD-1 and LAG-3 on CD4^+^ T cells (Butler et al., 2012). The combined blockade of PD-L1 and LAG-3 inhibitory molecules with antibodies, during *P. yoelii* and *P. chabaudi* malaria in mice accelerated clearance of parasitemia (Butler et al., 2012). This dual blockade of PD-L1 and Lag-3 improved CD4^+^ follicular T helper cell (T_FH_) numbers which correlated with enhanced antibody-mediated immunity (Butler et al., 2012). Moreover, infected mice treated with the anti-malarial drug chloroquine at day 8 and 9 post-infection, showed a lower level of CD4^+^ T cell dysfunction (Butler et al., 2012). These studies showed that lymphocyte exhaustion modulated immunity against malaria.

Subsequent studies used mice with a deletion of PD-1 (PD-1KO) to conclusively determine if PD-1 had a role in modulating immunity, given that PD-L1 can interact specifically with both B7-1 (Butte et al., 2007) and PD-1 (Iwai et al., 2003) to inhibit T cell activation. *P. chabaudi* malaria was investigated as this infection develops into chronic infections. It was shown that PD-1 mediated a reduction in the capacity of parasite-specific CD4^+^ T cells to proliferate and secrete IFN-γ and TNF-α during the chronic phase of malaria (day 35) indicating exhaustion of these cells (Horne-Debets et al., 2013). However, in contrast to the combined PD-L1/Lag-3 blockade study, no changes to T_FH_ numbers were observed. One likely explanation for this apparent contradiction is that PD-1 KO mice compared with wild type (WT) mice had a significantly higher proportion of regulatory T follicular cells (T_FR_ cells; Horne-Debets et al., 2013). T_FR_ cells are known to be suppressive *in vitro* and to limit the numbers of T_FH_ cells and germinal centers (GC) B cells *in vivo* (Linterman et al., 2011). Alternatively, since PD-L1 can also interact specifically with B7-1 to inhibit T cell activation (Butte et al., 2007), this pathway may control T_FH_ numbers in PD-1 KO mice.

## B CELLS AND EXHAUSTION

Antibodies are known to have a key role in controlling blood-stage infections (Cohen et al., 1961). Investigations into the mechanism of protection, found mice deficient in mature B cells developed a chronic relapsing parasitemia, confirming the need for antibodies to control chronic malaria (von der Weid et al., 1996). For antibodies to be protective, they have to undergo processes of class switching, somatic mutation, and affinity selection within GC where antibodies of the highest affinity are generated and selected. The formation of GC requires T_FH_ which also contribute to B cell differentiation into plasma and memory cells (Crotty, 2011).

The combined blockade of PD-L1 and LAG-3 increased numbers of CD4^+^ T_FH_ and GC B cells along with higher antibody titers which contributed to better control of blood stage of malaria (Butler et al., 2012). In contrast, PD-1 KO mice showed no significant improvement in numbers of germinal center B cells, plasma cells or antibody titers (Horne-Debets et al., 2013). The absence of any improvement in B cell function in PD-1KO mice could be explained by an absence of improvement in T_FH_ numbers or inhibitory signals to LAG-3 expressed on B cells (Kisielow et al., 2005).

## EXHAUSTION OF CD8^+^ T CELLS DURING MALARIA

PD-1-mediated cellular exhaustion has been best associated with exhaustion of CD8^+^ T cells. However, as described earlier, a role for CD8^+^ T cells in the clearance of blood-stage malaria is not widely acknowledged although their role in pathogenesis of cerebral malaria and damage to splenic architecture (Beattie et al., 2006) are known. Critically, PD-1 was recently shown to mediate a 95% loss in the numbers and functional capacity of parasite-specific CD8^+^ T cells during the acute phase of malaria, which exacerbated the infection leading to chronic malaria (Horne-Debets et al., 2013). This study examined the progression of chronic malaria in PD-1 KO mice compared to WT where 100% of mice develop chronic infections. Interestingly, <30% of the PD-1 KO mice developed chronic infections, and parasitemia levels in these mice were >100-fold lower than those in the WT mice. However, depletion of CD8^+^ T cells in PD-1 KO mice, increased peak parasitemia by 2-fold and 100% of the PD-1 KO mice developed chronic malaria (Horne-Debets et al., 2013). Overall, PD-1-mediated 80% reduction in numbers of tetramer^+^CD8^+^CD62L^-^ T cells and 95% reduction in capacity of CD8^+^ cells to proliferate in response to parasites, during the chronic phase of malaria (Horne-Debets et al., 2013). Of particular note is that even though PD-1 KO mice had more functional CD4^+^ T cells than WT mice and similar titers of parasite-specific antibodies, they still developed chronic malaria if CD8^+^ T cells were depleted. Finally, PD-1 KO mice had more granzyme B-expressing CD8^+^ T cells than WT mice suggesting that cytotoxic-killing of infected cells was involved. These observations highlight the crucial role of CD8^+^ T cells in protection against chronic malaria. In contrast, a previous study had found blockade of PD-L1 augmented experimental cerebral malaria which is mediated by pathogenic CD8^+^ T cells (Hafalla et al., 2012), indicating the pathway protects against cerebral malaria. The clinical significance of these findings are highlighted by studies in Kenya which found human CD8^+^ T cells from individuals infected with malaria, express PD-1 (Illingworth et al., 2013). Thus the role of CD8^+^ T cells requires particular consideration as it may explain why despite years of exposure to intense *Pf* transmission there was no evidence of acquired, sterile immunity (Tran et al., 2013). It may be that antibodies and CD4^+^ T cells provide protection against symptomatic malaria but CD8^+^ T cells are required for sterile immunity. Thus with PD-1 mediated exhaustion of CD8^+^ T cells, sterile immunity is never acquired as recently reported (Tran et al., 2013).

## CONCLUSION

Antibodies and CD4^+^ T cells are known to protect against blood-stage *Plasmodium* spp. infections. However, there is a growing body of evidence that show that CD8^+^ T cells have a role in protection. Furthermore, there is also increasing evidence that lymphocyte exhaustion mediates loss of protection by CD4^+^ and CD8^+^ T cells. Finally, studies have shown MBC numbers (Weiss et al., 2010) and antibody (Crompton et al., 2010) titers specific for the parasite decline following malarial infections. Accordingly, the likelihood of making a malaria vaccine is declining. The new frontier may be the development of therapeutics to block *Plasmodium*-mediated pathogenesis.

## Conflict of Interest Statement

The authors declare that the research was conducted in the absence of any commercial or financial relationships that could be construed as a potential conflict of interest.
